# AI-Simulated Patients for Training Shared Decision-Making: Feasibility Study in Medical Education

**DOI:** 10.2196/100467

**Published:** 2026-07-16

**Authors:** Lea Herschbach, Teresa Festl-Wietek, Alessandra Sonanini, Friederike Holderried, Tobias Albrecht, Bernd Herrmann, Anne Herrmann-Werner

**Affiliations:** 1 TIME - Tuebingen Institute for Medical Education University of Tübingen Tuebingen, Baden-Wurttemberg Germany; 2 Department of Otolaryngology, Head and Neck Surgery University Hospital Tübingen Tuebingen, Baden-Wurttemberg Germany; 3 Deutsches Zentrum für Psychische Gesundheit, Partner site Tübingen Tuebingen, Baden-Wurttemberg Germany

**Keywords:** artificial intelligence, chatbots, communication, medical education, patient participation, professional competence, SDM, self-efficacy, shared decision-making, simulation training

## Abstract

**Background:**

Shared decision-making (SDM) is a key element of patient-centered care; however, opportunities for structured and scalable SDM training remain limited in both medical education and clinical practice. Advances in AI have enabled chatbot-based simulations that may support repeated practice and provide automated feedback on SDM-related communication behaviors.

**Objective:**

This study aimed to (1) evaluate the agreement between AI-generated and human ratings of SDM performance, (2) compare SDM-related performance and attitudes between medical students and physicians, and (3) explore changes in communicative self-efficacy (CSE) following chatbot-based SDM practice.

**Methods:**

We conducted a feasibility study using a pre-post mixed methods design. Medical students and physicians practiced SDM in a 20-minute chat-based interaction with an AI-simulated patient, followed by automated feedback on SDM performance. Prior to the interaction, participants’ attitudes toward SDM (IcanSDM) and their CSE (Self-Efficacy in Patient-Centeredness Questionnaire [SEPCQ-24-GER]) were assessed; CSE was reevaluated after the intervention. SDM performance was measured using the validated Observing Patient Involvement in Decision Making (OPTION-12) scale, with the AI evaluating participants’ performance from the patient’s perspective. These AI-generated ratings were then compared with human ratings to evaluate agreement and rating quality. To further investigate discrepancies between AI and human ratings, a qualitative analysis of selected cases was conducted. Additional measures included demographics, perceived authenticity, and benefits of the interaction. Quantitative analyses included group comparisons, pre-post analyses, and psychometric evaluation of the OPTION-12 scale in an AI-mediated setting. The study was preregistered on the Open Science Framework.

**Results:**

A total of 58 participants were included in the analysis, 22 (37.9%) of whom were students, 34 (58.2%) were physicians, and 2 did not declare. Both groups reported similarly positive attitudes toward SDM, but medical students (n=22; mean 28.67, SD 4.32) achieved higher OPTION-12 performance scores than physicians (n=34; mean 22.79, SD 8.14). CSE showed a slight decrease following the intervention. Agreement between AI-generated and human OPTION-12 total scores was high (intraclass correlation coefficient=0.86, *df*=2,1; *P*<.001), although AI ratings were systematically higher. The OPTION-12 scale in the AI setting demonstrated good internal consistency (Cronbach α=0.88; mean interitem correlation *r*=0.43). Participants perceived the AI-simulated interaction as authentic and potentially useful for SDM training.

**Conclusions:**

This feasibility study provides preliminary and exploratory insights into the use of AI-simulated patients for SDM training in medical education. The findings contribute to the contextual validation of the OPTION-12 scale in AI-mediated consultations and provide valuable insights into participants’ attitudes toward SDM. However, while findings suggest potential for supporting communication skills development and structured feedback, conclusions are limited by the study design and sample size. Further research with larger samples and controlled designs is needed to establish effectiveness and generalizability.

## Introduction

### Background and Relevance of Shared Decision-Making

Shared decision-making (SDM) is a collaborative, patient-centered approach in which health care professionals and patients jointly make medical decisions based on evidence and patients’ preferences [1]. While SDM is often regarded as a modern model of care, its conceptual roots can be traced back to early discussions in the 1950s emphasizing patient involvement as a core element of the “modern doctor” [2]. Charles et al [3] later formalized SDM into a significant framework comprising 4 key elements: the involvement of at least 2 people, reciprocal information exchange, joint deliberation regarding treatment options, and agreement on a specific decision. SDM is widely recognized as a strategy to reduce informational and power asymmetries in clinical encounters by strengthening patient autonomy and encouraging active participation [4,5]. Empirical evidence indicates that SDM strengthens the therapeutic alliance and is associated with higher patient satisfaction, improved adherence, and better health outcomes [6-10] without increasing consultation time or health care costs [11]. Additionally, when patients experience decision-making as participatory, they report enhanced affective and cognitive outcomes, including greater perceived control, improved understanding, and increased emotional security [12]. SDM is particularly relevant in the management of chronic or complex conditions, where sustainable treatment agreements are essential [13]. Overall, SDM contributes to the quality, sustainability, and acceptance of medical decisions.

### Implementation Challenges in Clinical Practice

Despite its strong ethical and policy endorsement, SDM is not yet consistently implemented in routine clinical practice [14]. Although physicians often report favorable attitudes toward SDM, their actual engagement varies depending on the clinical context, the type of treatment decision, and patient characteristics [15]. Patient-related factors, including cultural beliefs, language barriers, and socioeconomic status, may further limit the consistent application of SDM in everyday care [6]. Moreover, effective participation requires both adequate knowledge and a sense of agency, which not all patients are equally equipped with [16]. Consequently, SDM remains widely endorsed in principle yet difficult to implement consistently in practice due to structural, contextual, and individual barriers [17].

### AI in Medical Education and Communication Training

Training programs aimed at improving SDM and communication skills vary considerably in content, structure, and delivery format, and evidence regarding their effectiveness remains limited [18]. While educational interventions for medical students and health care professionals can improve communication skills in the short term [19], robust evidence on long-term effects, participant satisfaction, and sustained behavioral change remains limited [20]. While patient-reported experience measures exist, they are subject to bias, exhibit low response rates, and are not widely implemented [21]. Observer-based measures rely on human raters and are therefore resource-intensive, prone to lapses in concentration, and yield only modest reliability [22-24]. In parallel, AI is increasingly being integrated into medical education, particularly in the domains of communication training, simulation-based learning, formative feedback, and reflective practice [25-27]. AI-driven systems, including large language models, enable scalable and standardized simulated patient encounters that allow learners to repeatedly practice clinical communication in a safe environment [28-30]. Such systems can also provide immediate, structured feedback, potentially supporting deliberate practice and self-regulated learning [31].

Recent studies suggest that large language models (eg, generative pretrained transformer [GPT]–based systems) can reliably evaluate communication quality in simulated interactions and distinguish between different levels of performance [24]. For example, Selvaraj et al [24] demonstrated that AI-generated assessments of SDM-related communication show strong correlations with human ratings, indicating potential for more scalable and efficient evaluation processes. However, despite these promising developments, empirical evidence on the educational impact of AI-based SDM training—particularly regarding learner outcomes such as communicative self-efficacy (CSE), attitudes, and behavioral change—remains limited. Furthermore, CSE as a key factor for effective communication and the implementation of SDM has not been addressed in this context so far. However, evidence shows that communication skills training can improve both performance and self-efficacy [32].

### Research Gaps and Study Rationale

Several important gaps remain in the current literature. First, there is limited evidence on the use of AI-simulated patients specifically for SDM training in medical education. Second, although AI systems have shown promise in evaluating communication behavior, few studies have systematically validated AI-generated feedback against human expert ratings in SDM contexts. Third, there is limited evidence on how AI-based SDM training influences CSE as an important learner-centered outcome in communication skills development. Finally, little is known about how learners perceive AI-generated feedback in SDM training and whether such feedback is experienced as useful and credible for supporting reflective learning.

To address these gaps, this study investigates a chatbot-based SDM training intervention using an AI-simulated patient combined with automated feedback. In addition, the accuracy and perceived usefulness of AI-generated feedback in SDM training are assessed. Furthermore, the study examines changes in CSE and compares SDM attitudes and performance between medical students and physicians.

To evaluate the effectiveness of the proposed intervention, the following research questions are examined:

- Research question 1: to what extent can the AI chatbot provide accurate feedback on SDM using the Observing Patient Involvement in Decision Making (OPTION-12) scale compared with human ratings?
- Research question 2: how do medical students and physicians differ in SDM attitudes and performance?
- Research question 3: how does CSE change after practicing SDM with an AI chatbot?

## Methods

### Study Design and Overview

This feasibility study used a mixed methods pre-post design to evaluate an AI-supported SDM training intervention. The study consisted of a single-session interaction with an AI-simulated patient, followed by automated feedback and postintervention assessment. In addition, a group comparison was conducted between licensed physicians and medical students. Quantitative data were collected at baseline and immediately after the intervention, complemented by qualitative reflections.

### Setting and Participants

Participants included medical students and licensed physicians. Eligibility criteria comprised sufficient proficiency in the German language and current enrollment in medical training after passing the second state examination or active clinical practice. Participants were recruited either via a study link distributed through mailing lists and professional networks or in person during psychosomatic training sessions, where they completed the study on site. Participation was voluntary, and no compensation was provided. Datasets with incomplete data and chats comprising fewer than 5 messages were excluded from analyses.

### Intervention and Procedure

The data collection took place from November 2025 until February 2026. Initially, participants were introduced to an explanatory flowchart [33] illustrating 6 steps of SDM to ensure a basic level of SDM knowledge across all participants (Multimedia Appendix 1). Participants then engaged in a standardized 20-minute text-based consultation with an AI-simulated patient designed to elicit SDM behaviors. The AI system was configured to assume the role of a patient with a predefined clinical case and responded dynamically to participant input while maintaining consistency across interactions through structured prompting. Following the consultation, participants received AI-generated structured feedback on their SDM performance. The entire process, consisting of chat and questionnaires, took about 30 to 40 minutes. Data collection occurred at 2 time points:

- Preintervention: attitudes toward SDM (IcanSDM) and CSE (Self-Efficacy in Patient-Centeredness Questionnaire [SEPCQ-24-GER])
- Postintervention: CSE, perceived authenticity, perceived usefulness of feedback, and demographic variables

**Figure 1:**
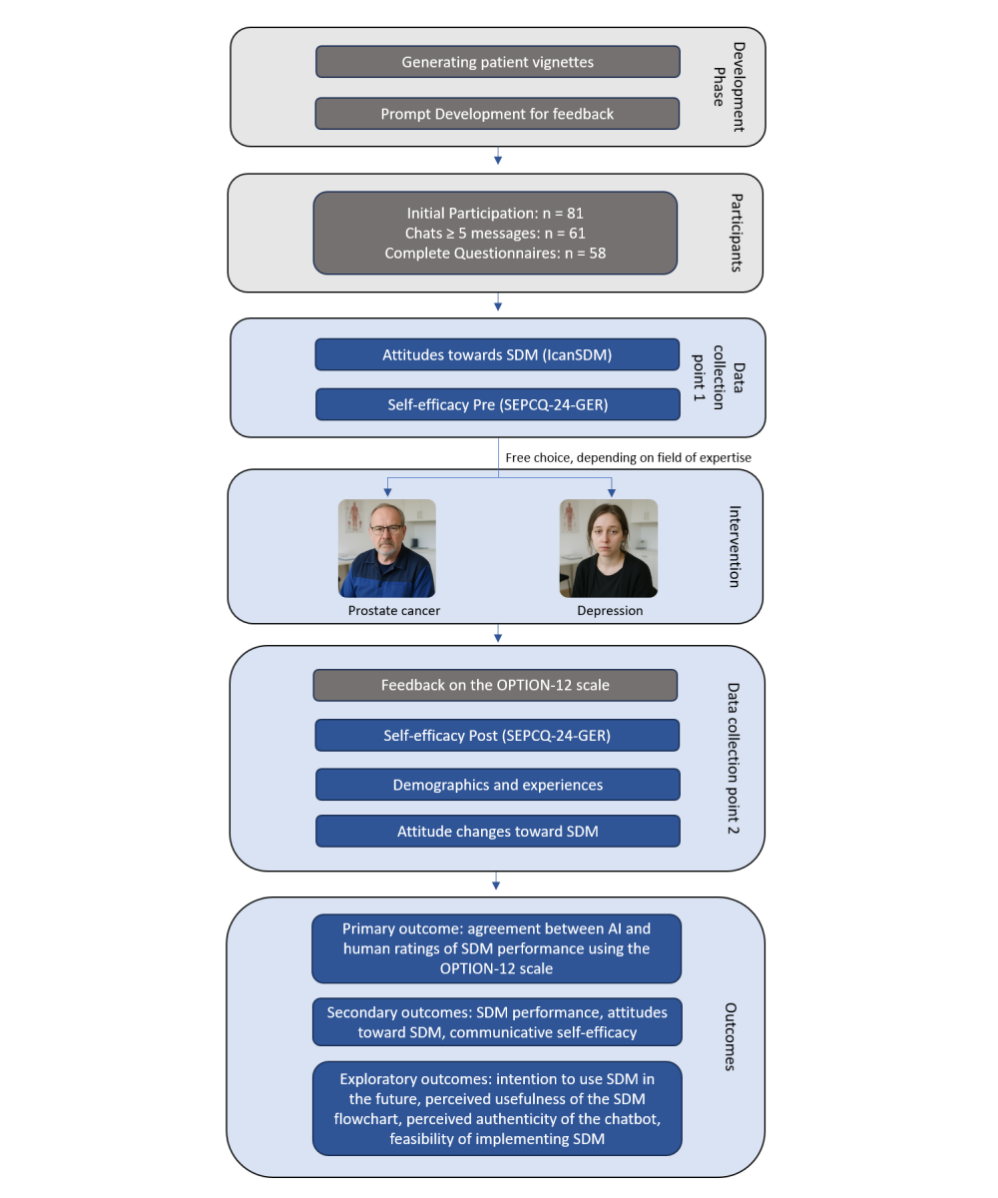
Flowchart illustrating the design of the experiment. OPTION-12: Observing Patient Involvement in Decision Making; SDM: shared decision-making; IcanSDM: measure of attitudes toward SDM; SEPCQ-24-GER: Self-Efficacy in Patient-Centeredness Questionnaire.

A detailed flowchart illustrating the study procedure is presented in Figure 1.

### AI-Simulated Patient and Feedback System

This application is described in detail in previous studies [34-36]. As validated in [35], this setup produces stable, medically accurate conversations. The model GPT-4-0613 was used via the OpenAI API with the temperature set to 0.1 and other parameters left at default. Details about models and parameters are provided in Multimedia Appendix 2. The feedback system was set to request feedback on buckets of 4 items during each model call. A general description of the 5 response levels of the OPTION-12 scale was included in the prompt, as well as the complete chat. The model GPT-5 was used via the OpenAI API as it was the frontier model at the time of evaluation. Multimedia Appendix 2 provides complete prompts, as well as models and parameters. Screenshots, exemplary interactions with the AI-simulated patient, and AI-generated feedback are provided in Multimedia Appendices 1 and 3 to illustrate the training experience and feedback structure.

To enable AI-generated feedback, a training dataset consisting of 9 expert-evaluated chats assessed using the OPTION-12 scale was developed in a separate pretest phase. These pretest data were collected prior to the main study with different participants and were fully independent of the final evaluation dataset. This strict separation ensured that no overlap occurred between data used for model refinement and data used for evaluation, thereby minimizing the risk of data leakage or circular evaluation. The absolute agreement between the human and AI ratings was moderate (intraclass correlation coefficient [ICC]=0.633, *df*=2,1, 95% CI 0.039-0.902; *P*=.02) but considered sufficient for the study’s purpose. The pretest annotations were used to support prompt development and calibration of the AI feedback system. The resulting prompts were iteratively refined through a structured manual optimization process, including the integration of illustrative examples and clarification of category boundaries to improve output consistency and interpretability. The final set of prompts is provided in Multimedia Appendix 2.

### Clinical Case Vignettes

Two clinical scenarios (prostate cancer and depression) were selected to represent distinct SDM contexts. The patient vignettes were specifically developed by experts. The first case describes a female student aged 28 years with recurrent depressive disorder. She is afraid of medication but is open to psychotherapy. The second case depicts a craftsman aged 62 years who presented with prostate cancer. He “just wants the thing out” and is skeptical of other procedures. The diagnosis is already known to the patients; the following conversation should therefore focus on discussing treatment options. The detailed patient vignettes are included in Multimedia Appendix 4.

Both cases involve distinct but comparable SDM challenges, including emotional burden, treatment uncertainty, and the need to balance medical evidence with patient preferences. The scenarios were also chosen to allow participants to engage with a case that was broadly aligned with their clinical background or expertise. Both cases were developed based on established clinical guidelines and structured to ensure comparability across participants. To ensure clinical validity and relevance, the cases were subsequently reviewed by additional experts in psychiatry and oncology. These experts assessed the medical accuracy, internal consistency, and appropriateness of the scenarios for SDM training. Feedback from this review process was incorporated to refine and finalize the case vignettes. The cases were integrated into the chatbot and comprehensively tested.

### Measures

#### Attitudes Toward SDM

Attitudes toward SDM were collected using the German version of the IcanSDM scale [37]. The scale consists of 8 items and examines the perceived barriers and attitudes of health care professionals toward SDM. The response scale is based on an 11-point Likert scale ranging from 0 (strongly disagree) to 10 (strongly agree).

#### CSE

CSE was measured using the subscale “sharing information and power” of the SEPCQ-24-GER [38]. The subscale assesses the extent to which respondents feel able to share information and power with patients. The subscale consists of 10 items and is answered on a Likert scale from 1 (to a very small extent) to 5 (to a very large extent). In the study, participants answered the scale twice, once before and once after the chat.

#### SDM Performance

To assess SDM performance, the OPTION-12 scale [22] was used. The OPTION-12 scale is an observer-based instrument consisting of 12 items that capture key elements of SDM during clinical encounters. Each item is rated on a Likert scale from 0 (competence not observed) to 4 (competence meets a very high standard), resulting in a total score reflecting the extent to which clinicians involve patients in decision-making. In this context, the AI adopted the role of the patient and evaluated the extent to which the patient was involved in the treatment decision using this instrument. The OPTION-12 scale is widely regarded as a valid and established instrument for assessing SDM in clinical encounters. The interrater ICC (0.77) and Cronbach α (0.68) are above acceptable thresholds. However, its applicability in AI-mediated consultation settings has not yet been sufficiently evaluated, as assessed in the present study. Our AI-generated OPTION-12 ratings demonstrated similar reliability, with Cronbach α=0.88 and single-rater absolute ICC=0.86. In addition to AI-generated ratings, an expert in clinical communication independently rated all anonymized transcripts. The expert was familiar with observer-based SDM assessment and received standardized instruction on the use of the OPTION-12 scale. Both AI and human ratings were conducted blinded to each other’s scores. No adjudication of discrepancies was performed. Agreement was assessed statistically using ICC and correlation analyses.

#### Perceived Feedback Quality and Authenticity

Participants rated the authenticity of the AI-simulated patient, the applicability of SDM in clinical practice, and their intention to use SDM more frequently. All items were assessed using a 5-point Likert scale ranging from “strongly disagree” to “strongly agree.”

### Outcome Definitions

#### Primary Outcome

The primary outcome was the level of agreement between AI-generated and human ratings of SDM performance as assessed using the OPTION-12 scale. Agreement was quantified using ICCs and interpreted as an indicator of the validity of AI-generated feedback.

#### Secondary Outcomes

Secondary outcomes included SDM performance as measured by the OPTION-12 total score, attitudes toward SDM assessed with the IcanSDM questionnaire, and CSE measured using the SEPCQ-24-GER. CSE was assessed preintervention and postintervention to evaluate changes following the AI-supported SDM training. Additionally, differences between medical students and physicians were analyzed for SDM performance and attitudes.

#### Exploratory Outcomes

As exploratory outcomes, participants rated their intention to use SDM more frequently in future clinical practice, as well as the perceived usefulness of the SDM flowchart for supporting future application. In addition, they assessed the perceived authenticity of the chatbot interaction and the feasibility of implementing SDM in everyday clinical practice. To further investigate discrepancies between AI and human ratings, a qualitative analysis of selected cases was conducted.

### Statistical Analysis

Chat scripts and questionnaire results were collected anonymously via the application. In total, 61 chats and 58 questionnaires were included in the analysis. Chats were segmented into question-answer pairs and compiled in a Microsoft Excel 2408 sheet [39]. Statistical analyses were performed in R (version 4.4.1; R Core Team, 2024 [40]), with significance set at *P*<.05. Figures were created using Microsoft Excel and R. All statistical tests were conducted using a 2-sided significance level of α=.05. Descriptive statistics were calculated for all variables. Reported metrics include means, SDs, percentages, and Pearson correlations.

SDM performance was operationalized using the total score of the OPTION-12 scale. To evaluate the applicability of the OPTION-12 scale in the AI setting, internal consistency was assessed using Cronbach α, and item-level relationships were examined using interitem correlations. To assess the quality of AI-generated feedback, OPTION-12 ratings produced by the AI were compared with human ratings of the same interactions. Agreement between raters was evaluated using ICCs and Pearson correlation. ICCs were interpreted according to established guidelines [41]. Based on the 95% CI of the ICC estimate, values below 0.50, between 0.50 and 0.75, between 0.75 and 0.90, and above 0.90 indicate poor, moderate, good, and excellent reliability, respectively. These thresholds were used to interpret the level of agreement between AI-generated and human ratings.

Normality of continuous variables was assessed prior to analysis. Nonparametric Wilcoxon signed-rank tests were used when normality assumptions were violated. Homogeneity of variance between groups was assessed using variance ratio tests (*F* test). Depending on the outcome, either Student *t* test for independent samples (in cases of equal variances) or Welch *t* test (in cases of unequal variances) was applied to calculate group differences. Changes in CSE (pre-post) were analyzed using the Wilcoxon signed-rank test, as the distribution of paired differences deviated from normality. In addition to inferential statistics, the effect size was estimated using Cliff delta, a nonparametric measure that quantifies the magnitude and direction of differences between groups by estimating the probability of stochastic dominance without assuming a normal distribution.

### Qualitative Analysis

A qualitative analysis of selected cases was conducted to investigate differences between AI and human OPTION-12 scores. Chats with the largest absolute differences in the OPTION-12 total scores were identified and subjected to in-depth examination. For each selected chat, item-level ratings were compared, and those with the greatest divergences were analyzed in detail. The analysis was guided by the OPTION-12 coding manual [42], with particular attention to how rating criteria were applied. This approach allowed for a systematic exploration of underlying reasons for rating differences between AI and human raters.

### Preregistration

The study design, hypotheses, and analysis plan were preregistered on October 23, 2025, prior to data collection on the Open Science Framework [43].

### Ethical Considerations

The study was approved by the Ethics Committee of the Faculty of Medicine at University Hospital Tübingen (375/2025BO2). Participation was voluntary, and no compensation was offered for participation. Informed consent was obtained from all participants prior to participation. Participants confirmed their agreement to the data collection and processing by ticking the respective box on the computer. Participants were fully informed about the study’s purpose and procedures. All data were anonymized. Appropriate measures were taken to ensure the confidentiality and security of participant information. All procedures were conducted in accordance with the Declaration of Helsinki and ethical guidelines for human participants.

## Results

### Overview

Results are presented according to participant-level and encounter-level analyses and structured in line with the 3 research questions. All analyses were conducted in accordance with the prespecified primary, secondary, and exploratory outcomes.

### Participant Characteristics and Outcomes

The following results address research questions 2 and 3 at the participant level.

#### Demographics

Of the 81 individuals who accessed the study in the first place, datasets with incomplete responses or fewer than 5 chat messages were excluded, as they were considered to reflect insufficient engagement. The final sample consisted of 58 participants, of whom 36 identified as female, 20 as male, and 2 did not specify. A total of 22 participants were students in their practical year (mean age 28, SD 3.08 years), 34 participants were certified physicians (mean age 35.79, SD 6.51 years), and 2 did not declare. Further, 27 participants reported working in a hospital setting, while 15 were working in private practice; the rest did not specify. Regarding medical specialty, 20 participants reported a background in general medicine, 4 in gynecology, 1 in psychosomatic medicine, and 1 in pediatrics; the remaining participants selected “other.”

#### Differences Between Medical Students and Physicians

In line with the secondary outcomes, a comparison of SDM attitudes between students (n=22) and physicians (n=34) was conducted using the IcanSDM mean score. Only participants who completed the IcanSDM questionnaire and specified their background (student or physician) were included in the analysis (n=56). As SDM scores were normally distributed for both students (W=0.974; *P*=.79) and physicians (W=0.940; *P*=.06) and no significant difference in variances (*F*_21,33_=0.645; *P*=.29) was found, a standard independent-samples 2-tailed *t* test was conducted. The results indicated no significant difference in SDM attitudes between students (n=22; mean 3.96, SD 1.05) and physicians (n=34; mean 4.13, SD 1.31; t_54_=–0.507; *P*=.61; 95% CI –0.835 to 0.498).

In addition, encounter-level SDM performance, measured by the OPTION-12 total score, was compared between students (n=22) and physicians (n=34). In total, 56 participants were included in this analysis. The OPTION scores were normally distributed for both students (W=0.955; *P*=.40) and physicians (W=0.962; *P*=.28). The assumption of equal variances was violated (*F*_21,33_=0.290; *P*=.004); therefore, a Welch 2-tailed 2-sample *t* test was conducted. Results showed that students (n=22; mean 28.41, SD 4.38) performed significantly better than physicians (n=34; mean 22.79, SD 8.14; t_52.60_=3.49; *P*=.002; 95% CI 2.24-8.99).

#### Changes in CSE Following SDM Practice

In the next step, also in line with the secondary outcomes, changes in CSE before and after practicing SDM in the chat were examined using the SEPCQ-24-GER scores (n=58 participants). A Wilcoxon signed-rank test was conducted as the differences between pretraining and posttraining scores were not normally distributed (W=0.865; *P*<.001). Results showed a statistically significant change in CSE (V=216; *P*=.003) with higher scores before training (mean 4.02, SD 0.49) than after (mean 3.77, SD 0.79). In an additional post hoc directional analysis using a one-sided test (alternative=“less”), the decrease in scores remained statistically significant (V=216; *P*=.002). A small negative effect was confirmed by Cliff delta (Δ=–0.186; 95% CI –0.384 to 0.028).

#### Evaluation and Benefits

As exploratory outcomes, participants’ perceptions of the AI-supported SDM training were assessed. The analysis included 57 participants who completed the exploratory questions. Participants generally reported positive perceptions of SDM. For the intention to use SDM more often from now on, 52.63% (30/57) of participants agreed or strongly agreed, and 36.84% (21/57) were neutral. A total of 66.67% (38/57) of participants agreed or totally agreed with the statement that the flowchart depicting SDM shown in the study would help them apply SDM more often in the future. The chatbot was considered authentic by 54.39% (31/57) of participants, though 29.83% (17/57) were neutral. A total of 87.72% (50/57) of participants agreed or strongly agreed that SDM is feasible in everyday clinical practice.

### Encounter-Level Analysis of SDM Performance

#### Overview

The final section reports validation analyses of AI-generated OPTION-12 ratings (research question 1). For the validation analysis, 61 chats meeting predefined completeness criteria (minimum of 5 messages exchanged) were included to ensure meaningful participation.

#### Descriptive Results of OPTION-12 Scores

Overall, the mean OPTION-12 score across encounters was 25.23 (SD 7.34; n=61) out of a maximum of 48 possible points. The median score was 26 (IQR 21-30). Observed scores ranged from 7 to 37. At the item level, the lowest mean score was obtained on item 3 (“The clinician assesses the patient’s preferred approach to receiving information to assist decision-making”; mean 0.87, SD 0.74), and the highest mean score on item 2 (“The clinician states that there is more than one way to deal with the identified problem”; mean 3.23, SD 0.96).

#### Psychometric Properties of AI-Generated OPTION-12 Ratings

The AI-generated ratings (n=61) demonstrated good internal consistency across the 12 items (Cronbach α=0.88, 95% CI 0.83-0.92), with an average interitem correlation of *r*=0.43. The corrected item-total correlation (r.drop) ranged from 0.440 (item 12: “The clinician indicates the need to review the decision [or deferment]”) to 0.733 (item 2: “The clinician states that there is more than one way to deal with the identified problem”). The corrected item-total correlation (r.drop) reflects the correlation between each item and the total score excluding that item, indicating that all items positively contributed to internal consistency. More psychometric details for human and AI ratings are presented in Table 1.

**Table 1:** Observing Patient Involvement in Decision Making (OPTION-12) item-level statistics.

OPTION-12 items	Human rating, mean (SD)	AI rating, mean (SD)	Corrected item-total correlation (r.drop)	Interrater agreement (ICC)
Item1	2.77 (0.80)	2.97 (0.58)	0.717	0.578
Item2	2.61 (1.05)	3.23 (0.96)	0.733	0.645
Item3	0.41 (0.74)	0.87 (0.74)	0.560	0.520
Item4	2.49 (0.96)	2.49 (0.79)	0.713	0.787
Item5	2.05 (0.96)	1.90 (0.83)	0.713	0.727
Item6	2.44 (0.99)	2.43 (0.74)	0.445	0.689
Item7	2.08 (1.20)	2.16 (1.02)	0.521	0.769
Item8	1.46 (1.29)	1.33 (0.72)	0.663	0.609
Item9	1.74 (1.22)	2.11 (1.32)	0.488	0.839
Item10	1.59 (0.92)	1.69 (0.90)	0.714	0.724
Item11	1.92 (0.88)	2.07 (0.81)	0.614	0.536
Item12	1.64 (1.17)	1.98 (1.43)	0.440	0.791
Total	23.2 (7.52)	25.2 (7.34)	N/A^a^	0.859

^a^N/A: not applicable.

#### Agreement Between AI and Human OPTION-12 Ratings

**Figure 2:**
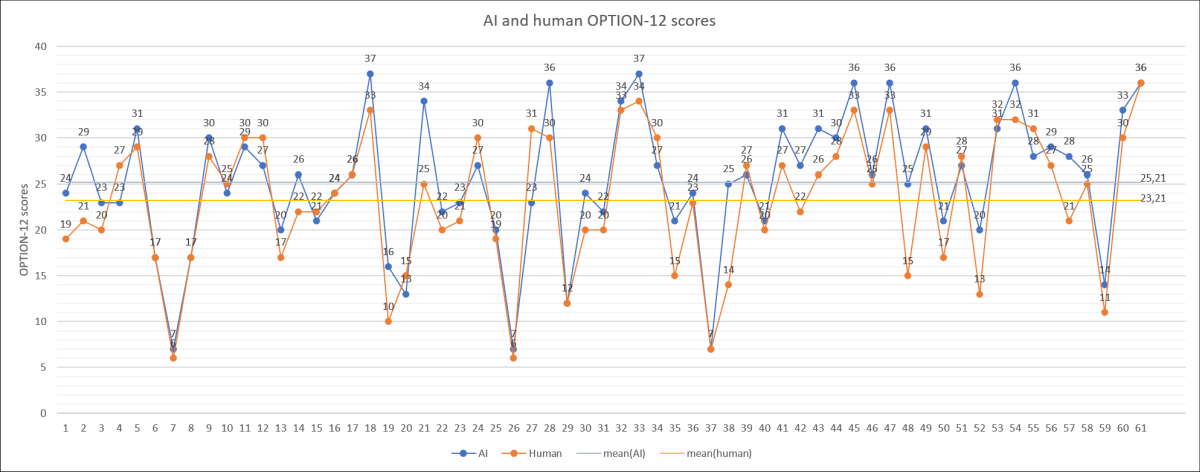
Comparison of human and AI ratings on the Observing Patient Involvement in Decision Making (OPTION-12) scale, including mean values.

In line with the primary outcome, agreement between AI and human ratings (n=61) was analyzed. There was strong agreement between the AI and the human rater for the total score (ICC=0.86, *df*=2,1; *P*<.001), although the AI systematically assigned slightly higher scores (mean 25.21, SD 7.34) than the human rater (mean 23.21, SD 7.51). The ICC quantifies the level of agreement between human and AI ratings. ICC (*df*=2,1) values per item, reflecting absolute agreement between AI and human ratings, ranged from 0.520 (item 3: “The clinician assesses the patient’s preferred approach to receiving information to assist decision-making”) to 0.839 (item 9: “The clinician offers the patient explicit opportunities to ask questions during the decision-making process”), suggesting moderate to strong agreement across items. Table 1 provides more details. The ratings of the 2 raters were strongly positively correlated (*r*=0.88), indicating a high level of consistency in relative scoring across cases. Exact agreement between raters occurred in 9.84% (6/61) of cases. In 21.31% (13/61) of cases, the human rater assigned a higher score, whereas in 68.85% (42/61) of cases, the AI assigned a higher score. Figure 2 provides more details.

### Qualitative Analysis of Individual Chats

As can be seen in Figure 2, the ratings that AI and human raters allocated differ considerably in some cases. To understand these large discrepancies, the 3 chats with the highest differences in the total OPTION-12 score (chats 21, 38 and 48 with differences of 9, 11, and 10, respectively) were analyzed qualitatively. For each chat, item-level differences between AI and human ratings were examined, and items with the greatest discrepancies were analyzed in detail and compared against the coding manual definitions [42]. In chat 21, item 5 (“The clinician explains the pros and cons of options to the patient”), the physician focused exclusively on explaining the effectiveness and side effects of medications rather than outlining the advantages and disadvantages of all available treatment options. While the human rater assessed the item as a minimal attempt, the AI rated it as exhibiting a good standard. In chat 38, item 7 (“The clinician explores the patient’s concerns [fears] about how problem[s] are to be managed”), AI ratings credited items as fulfilled even when patients introduced relevant information without being explicitly prompted by the physician. Similarly, for item 10 (“The clinician elicits the patient’s preferred level of involvement in decision-making”), the physician only implicitly inquired about patients’ preferred level of involvement in decision-making; nevertheless, the patient provided minimal indications of their preferences, which were again recognized as sufficient by the AI but not by the human rater. In chat 48, item 2 (“The clinician states that there is more than one way to deal with the identified problem”), the physician acknowledged the existence of multiple valid treatment approaches without providing a comprehensive or detailed overview. The AI rated this as “The behavior is observed and performed at a high level,” while the human rater rated it as “The clinician gives the impression that the options are valid and need to be examined more closely.” Overall, discrepancies appear to stem from differences in how implicit communication and patient-initiated contributions are interpreted and evaluated. More details are available in Multimedia Appendix 5.

## Discussion

### Principal Findings

This study examined a chat-based SDM training intervention using an AI-simulated patient combined with automated feedback on SDM performance. The principal findings indicate that participants were able to engage in structured SDM interactions within the AI-simulated patient and that the system was generally perceived as feasible for communication training. Preliminary results indicate good to strong agreement between AI-generated and human ratings of SDM performance based on the OPTION-12 scale. In addition, medical students and physicians reported similarly positive attitudes toward SDM, but students achieved higher performance scores on the OPTION-12 scale. CSE slightly decreased following the SDM practice intervention and feedback. The observed engagement with the AI chatbot in SDM tasks, as well as the alignment between AI and human ratings, suggests that large language models can support structured communication training. However, given the exploratory nature of the study, the limited sample size, and the fact that the results do not provide sufficient evidence for the validity of AI-generated assessments for SDM performance, the results should be interpreted as preliminary evidence for future research.

### Interpretation of the Findings

Both groups of medical students and licensed physicians reported similarly positive attitudes toward SDM. This is in line with the consistent endorsement of SDM principles across levels of professional training described in the literature [15]. In contrast, differences emerged in observed SDM performance, with medical students achieving significantly higher OPTION-12 scores than physicians, indicating greater adherence to structured SDM approaches in recently trained students [44]. These findings are in line with previous research reporting an inverse association between SDM-related competencies and age [45]. One possible explanation is that students may adhere more closely to structured SDM models due to recent formal training, whereas physicians may rely more on routinized communication patterns shaped by clinical experience [46-48]. The discrepancy between attitudes and observable behavior highlights the potential gap between endorsement of SDM and its practical implementation [14,15].

The observed decrease in CSE following the intervention may reflect a possible recalibration effect, as has been suggested in previous educational research [49]. This effect describes a phenomenon in which engagement in structured learning and feedback increases awareness of the complexity of the task and potentially lowers self-perceived competence. However, in the absence of a control group, this interpretation remains speculative, and causal conclusions cannot be drawn.

Descriptive analyses of OPTION-12 values showed moderate overall performance and values similar to those reported in the literature [50]. Certain SDM components appeared to be implemented more consistently (eg, “The clinician states that there is more than one way to deal with the identified problem”), while others were more challenging (eg, “The clinician assesses the patient’s preferred approach to receiving information to assist decision-making”).

Regarding the comparison of AI-generated and human ratings, the 2 measures showed a strong positive correlation and good interrater reliability, indicating a high level of consistency, as previously described in similar contexts [24]. Nevertheless, the AI consistently assigned higher scores than the human rater. This tendency to agree and give socially desirable answers is a pattern that is already recognized [51,52]. In the exemplary qualitative analysis conducted as part of our study, it became clear that discrepancies appear to stem mainly from differences in how implicit communication and patient-initiated contributions are interpreted. Overall, these findings should be interpreted cautiously given the exploratory nature of the study and the absence of a fully established validation framework.

### Practical Implications

The results suggest that AI-powered chatbots may be a viable and scalable tool for teaching SDM skills in medical education and professional development. Feedback on real clinical conversations is often difficult to implement, critical to the protection of personal data, and resource-intensive to obtain, making AI a less demanding alternative. This study shows that AI can apply the OPTION-12 scale in a similar way to a human rater and can adopt the patient’s perspective during the assessment. Without having surveyed actual patients, we were therefore able to gain an initial impression of the patient’s perspective, as suggested in the literature [20,21]. Such tools can complement existing communication training formats by providing low-threshold, time-independent practice opportunities and individualized feedback. In terms of training practice, integrating AI-supported SDM training into medical curricula could help standardize the teaching of SDM principles and establish SDM on a broad scale.

### Limitations and Recommendations for Future Research

Several limitations should be considered when interpreting the results of this study. First, the simulated consultations were text-based and conducted with an AI-simulated patient, which may not have fully captured the complexity of real-world clinical encounters, particularly nonverbal aspects of communication. Future research should therefore examine the transferability of SDM skills acquired in chatbot-based training to real-world patient encounters. Second, the study was based on a pre-post design without a control group, which limits causal inferences about the effects of chatbot-based SDM training. Future studies should focus on randomized controlled designs compared with traditional teaching methods. Furthermore, longitudinal follow-up assessments are necessary to evaluate sustained impact. Third, the study was conducted with a relatively small and self-selected sample, which may introduce selection bias and limit representativeness. Furthermore, the study did not assess participants’ prior familiarity with AI tools, digital literacy, or typing speed, all of which may have influenced their interaction with the chatbot. Participants with higher levels of digital competence or faster typing skills may have been better able to engage with the system, potentially confounding performance outcomes. Future studies should consider measuring and controlling for these factors to better isolate the effects of the intervention. The fairly high dropout rate could be attributed to a lack of time and competing clinical or academic obligations, since this was a voluntary study with no incentives. Participants may have clicked on the link out of initial interest but lost interest shortly thereafter, perhaps due to a lack of time. Future studies should therefore aim to recruit larger and more diverse samples across multiple institutions to improve generalizability and reduce selection bias. Fourth, the evaluation of the AI-generated feedback was conducted by only 1 rater. This introduces a potential risk of subjectivity and limits the robustness of the validation process, reducing the methodological rigor of the findings. Future research should therefore incorporate multiple raters and report interrater reliability metrics to ensure a more reliable and comprehensive validation of AI-generated feedback. Finally, performance bias cannot be excluded, as participants were not blinded to the study purpose and may have adapted their behavior based on perceived expectations. Similarly, assessment bias may have been introduced due to the lack of blinding in human ratings. Future research should implement blinding procedures for both participants and raters wherever feasible to minimize expectancy effects and rating bias.

### Conclusion

This exploratory study provides preliminary evidence that AI-simulated patients can be integrated into communication training to support SDM. Participants were able to engage in structured SDM interactions within the AI environment, and initial results suggest a good level of agreement between AI-generated and human ratings of SDM performance. However, these findings should be interpreted cautiously due to the exploratory design, limited sample size, and methodological constraints. While AI-simulated patients may have potential as a supportive tool for SDM training, further research is required to establish validity, reliability, and generalizability using larger samples and standardized, rigorously validated AI implementations.

## Appendix

Multimedia Appendix 1 Screenshots of the AI-based Application.

Multimedia Appendix 2 Model and prompting details.

Multimedia Appendix 3 Example of an interaction with the AI-simulated patient (translated into English).

Multimedia Appendix 4 AI-simulated patient vignettes.

Multimedia Appendix 5 Qualitative analysis.

## Abbreviations

CSE: communicative self-efficacy
GPT: generative pretrained transformer
ICC: intraclass correlation coefficient
OPTION-12: Observing Patient Involvement in Decision Making
SDM: shared decision-making
SEPCQ-24-GER: Self-Efficacy in Patient-Centeredness Questionnaire

## Data Availability

The data that support the findings of this study are available from the corresponding author (LH) upon reasonable request.
