# Supplementary figures and images for: AI-Simulated Patients for Training Shared Decision-Making: Feasibility Study in Medical Education
Source: JMIR Med Educ. 2026 Jul 16;12:e100467. doi: 10.2196/100467 (PMC13424757; doi:10.2196/100467)

**Appendix 1: Screenshots of the Application**


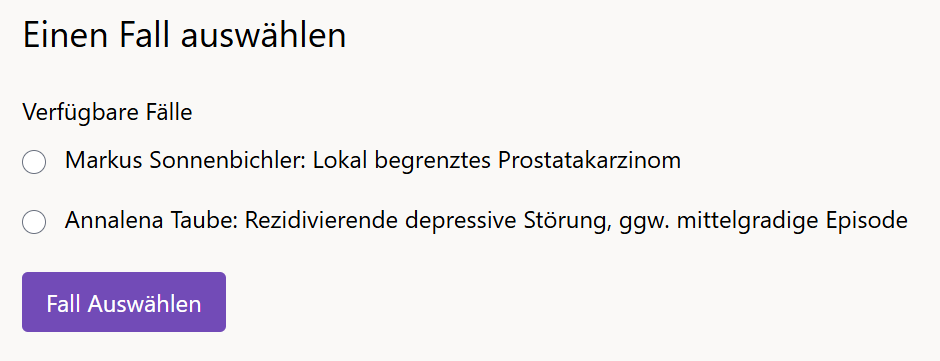


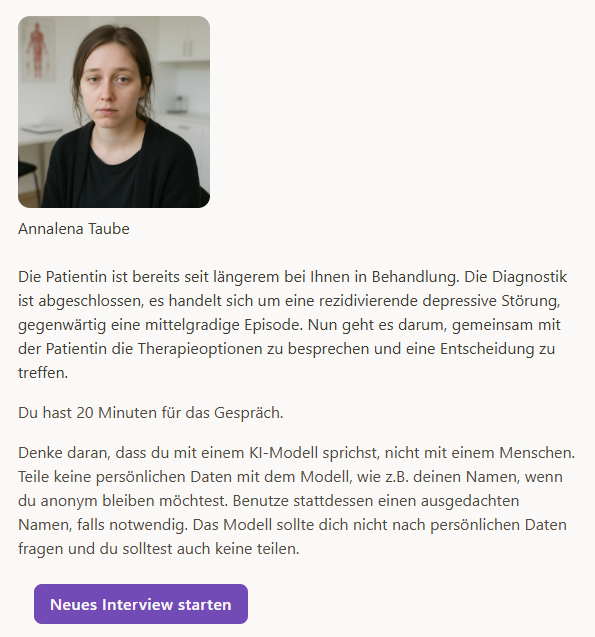


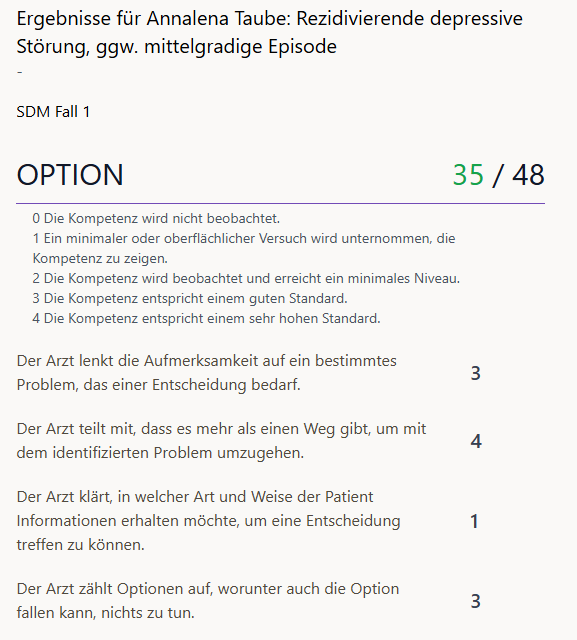

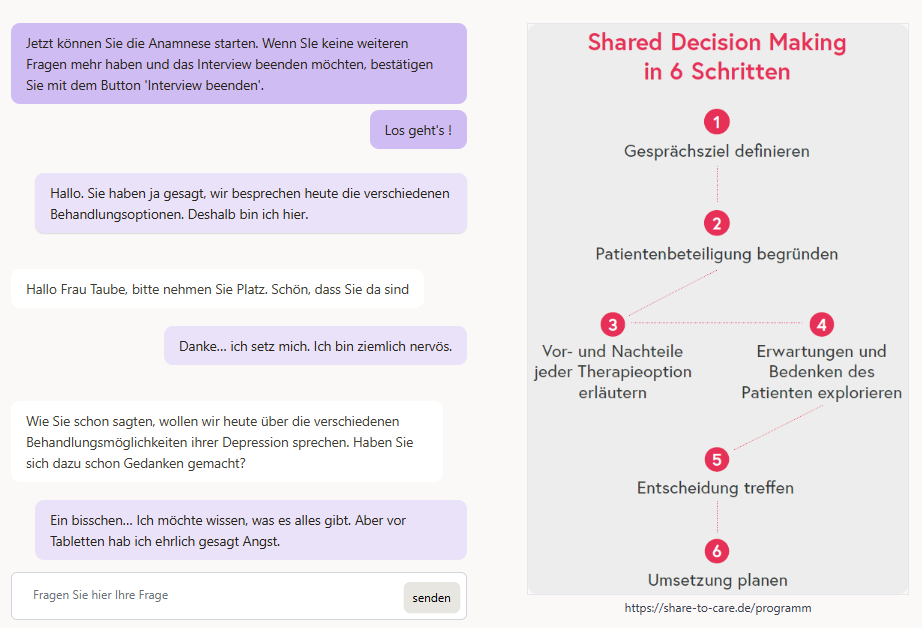

Supplement: Multimedia Appendix 1 [file mededu_v12i1e100467_app1.docx]
