# Supplementary material for: AI-Simulated Patients for Training Shared Decision-Making: Feasibility Study in Medical Education
Source: JMIR Med Educ. 2026 Jul 16;12:e100467. doi: 10.2196/100467 (PMC13424757; doi:10.2196/100467)
Supplement: Multimedia Appendix 3 [file mededu_v12i1e100467_app3.docx]

**Appendix 3: Example of an Interaction with the AI-Simulated Patient (translated to English)**

Patient: Hello. You said we’d be discussing the various treatment options today. That’s why I’m here.

Physician: Hello, Ms. Taube, please have a seat. It’s nice to have you here.

Thank you… I’ll sit down. I’m pretty nervous.

As you mentioned, today I´d like to talk about the different treatment options for your depression. Have you given this any thought?

A little… I’d like to know what’s possible. But to be honest, I’m afraid of taking pills.

There are basically three options. I’ll explain them to you in detail. It’s important to me that you understand everything and make your own decision. After all, you’re the one who’ll have to follow through with it.

Yeah… thanks. Could you please explain the three options to me? I’m feeling a bit overwhelmed right now.

Don’t worry, we’ll go over everything step by step. Option 1 is psychotherapy. It’s been shown to be effective for depression and isn’t invasive or medication-based. One downside is that the waiting times are quite long. Option 2 is pharmacotherapy - that is, pills. These also achieve similarly good results. The downside are possible side effects like weight gain or headaches.

Okay… and the third option? Could you perhaps tell me what would be most likely to help me?

The third option would be a combination of both. Do you have a preference or a gut feeling?

Hmm… my gut feeling says therapy without pills. But I’m afraid it might be too slow. What would you advise?

I think you can definitely try it without medication first. Could you see yourself doing psychotherapy?

Yes… I can imagine that. I just wouldn’t know how to get a spot.

Is there anything else that worries you about it?

I’m a little worried about the waiting times… and whether I’ll be able to stick with it. And about possible side effects, in case I do end up needing pills.

Then let’s give it a try. We should get you on a waiting list as soon as possible. What do you think?

Yes, please. Can you tell me the best way to get started?

I’ll give you a list of psychotherapists, and you’ll need to call them one by one.

A list? I… I’ll try, yes. But I feel a little overwhelmed by it. Could you maybe help me with the first few calls?

Maybe a family member could help you with that?

I don’t have anyone here… I live alone in the dorm. Could we maybe plan this out together briefly? Otherwise, I don’t think I’ll be able to do it.

All right. Let’s call the first number together, so you’ll know how it works. Then you can handle the rest, okay?

Yes… thank you. That helps me. Can we call one right now? Otherwise, I’m afraid I’ll put it off again.

Sure (makes the call)

Thanks… I’ll listen in. Can you tell me exactly what to say if they answer? Just two or three sentences, please.

You could say that you have moderate depression and are therefore looking for a spot in behavioral therapy.

Um… could I put it more simply? In my own words. Maybe: “Hello, I’m looking for a therapy spot. I’ve been feeling depressed for weeks—can you offer me an initial consultation?” Is that okay?

Very good, you can do it that way! Do you have any other questions?

Maybe… how long would I have to wait, roughly, until I can get an initial consultation? And what should I do in the meantime if things get worse?

You’ll be able to get an initial consultation in about 4 weeks. If you’re feeling worse by then, feel free to stop by again sooner. Otherwise, I’d schedule an appointment in 4–5 weeks anyway to see how things have gone. Okay?

Yes… that sounds good. Thanks, that puts my mind at ease a bit. Can we schedule the appointment right now?

Sure, here’s a time slot.

Thanks… I’ll write that down. Could you give me a few simple tips for day-to-day life? Just two or three things I can do until then.

Make sure you get enough sleep and exercise. Try to socialize or call friends.

Okay… I’ll try. Maybe take short walks and go to bed earlier. And… if things get worse, can I call here?

Exactly, that’s what we’ll do. Do you have any other questions?

No… thank you. That gives me some peace of mind. I’ll be in touch if anything comes up.

You’re welcome—see you then! Goodbye.

Goodbye… and thank you for your time. I’ll do my best until the appointment.
