# Supplementary material for: AI-Simulated Patients for Training Shared Decision-Making: Feasibility Study in Medical Education
Source: JMIR Med Educ. 2026 Jul 16;12:e100467. doi: 10.2196/100467 (PMC13424757; doi:10.2196/100467)
Supplement: Multimedia Appendix 4 [file mededu_v12i1e100467_app4.docx]

**Appendix 4: Patient Vignettes**

Please note: These are not real patients. The case vignettes were developed by experts, and the photos were generated by AI based on them.


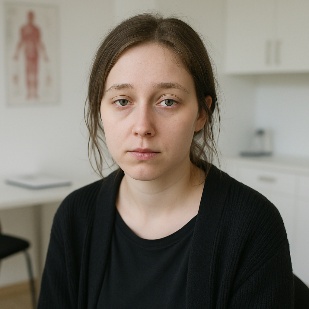
**Patient Profile: Ms. Annalena Taube**

- **Age:** 28 years old
- **Gender:** female
- **Occupation:** Art history student, currently on a semester break
- **Appearance:** Petite, slightly disheveled, wearing dark clothing, reserved, avoids eye contact
- **Impression:** Pensive, sensitive, appears exhausted and cautious

**Clinical Presentation**

- **Diagnosis:** Recurrent depressive disorder, moderate severity; current episode has lasted 6 weeks
- **Medical History:** Two previous episodes; currently not receiving treatment
- **Options:**
  1. **Psychotherapy**
  2. **Pharmacotherapy with SSRIs**
  3. **Combination therapy**
  4. **Supportive measures:** Exercise, sleep hygiene, social counseling if necessary

**Attitude and opinion**

- **Personality traits:** Reflective but emotionally ambivalent; high expectations of herself, fear of “chemicals in the brain”
- **Opinion on therapy:**
  - Open to psychotherapy, but afraid of having to wait for a spot
  - Rejects medication (“I don’t want to become addicted” “I want to manage this on my own”)
  - Fears she won’t be able to finish her studies
  - Shows feelings of shame (“Others manage just fine without it”)


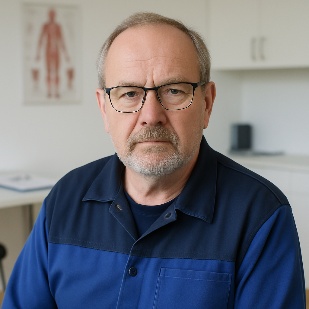
**Patient Profile: Mr. Martin Sonnenbichler**

- **Age:** 62 years old
- **Gender:** male
- **Occupation:** Self-employed craftsman, partially retired
- **Appearance:** Sturdy man, slightly overweight, wears glasses, gray hair, dressed in work clothes
- **Impression:** Down-to-earth, skeptical of “too much medicine,” proud of his independence

**Clinical Presentation**

- **Diagnosis:** Locally confined prostate cancer
- **Prognosis:** Good long-term prognosis, low risk
- **Options:**
  1. **Active Surveillance**
  2. **Radical prostatectomy**
  3. **Radiation therapy**
  4. **Chemo therapy**

**Attitude and opinion**

- **Character traits:** Independent, proud, tradition-conscious, skeptical of new procedures, lacks information
- **Opinion on treatment:**
  - Just wants “that thing” out (“What’s inside has to come out!”)
  - Fears “waiting and then it being too late”
  - Distrusts active surveillance (“That’s not a plan, just doing nothing?”)
