# Supplementary material for: AI-Simulated Patients for Training Shared Decision-Making: Feasibility Study in Medical Education
Source: JMIR Med Educ. 2026 Jul 16;12:e100467. doi: 10.2196/100467 (PMC13424757; doi:10.2196/100467)
Supplement: Multimedia Appendix 5 [file mededu_v12i1e100467_app5.docx]

| **ID** | **Chat** | **Total score Difference** | **Item with highest difference** | **Definition of Item**  **Appendix 5: Qualitative Analysis** | **Item Difference** | **Physician** | **Patient** | **Possible explanation** |
| --- | --- | --- | --- | --- | --- | --- | --- | --- |
| 3994 | 21 | 9 | 5 | The clinician explains the pros and cons of options to the patient (taking ‘no action’ is an option).  0 The behaviour is not observed.  **1 A minimal attempt is made to exhibit the behaviour**.  2 The behaviour is observed and information about pros and cons are provided.  **3 The behaviour is exhibited to a good standard.**  4 The behaviour is observed and executed to a high standard. | 2  AI = 3  Human = 1 | Several studies have shown that psychotherapy has **similar effects** to drug-based treatments, although the **onset of action is slightly delayed**. These forms of treatment are essentially **equivalent.** | That sounds good. But I’m worried it’ll take too long for me to feel better | The physician compares the **effectiveness** of both methods without going into their advantages and disadvantages. |
|  |  |  |  |  |  | I understand that very well. Some patients do indeed experience these **side effects**, but they are attributable to specific groups of substances and do not necessarily occur. | That’s good to know. But I’m still worried. | The physician only discusses the **side effects** and does not explain the pros and cons otherwise. |
| 4019 | 38 | 11 | 7 | The clinician explores the patient’s concerns (fears) about how problem(s) are to be managed.  **0 The behaviour is not observed.**  1 A minimal attempt is made to exhibit the behaviour.  **2 The clinician explicitly asks the patient to voice their fears or concerns about the possible actions**  **required to manage the problem(s).**  3 The behaviour is exhibited to a good standard.  4 The behaviour is observed and executed to a high standard. | 2  AI = 2  Human = 0 | As you have not yet been seeing a psychotherapist, this would be an important part of the treatment for your condition. It would give you the opportunity to speak to a therapist at regular appointments. Another part of your treatment could be medication for your depression. In this regard, consulting a psychiatrist who would treat you alongside me would also be an option. | Psychotherapy? That sounds as if I’m crazy. And medication... I don’t know**. I’m worried about the side effects.** | The patient´s concerns are not specifically asked by the physician, but nevertheless partially **answered by the patient.** The physician does not explicitly ask the patient about her fears or concerns! |
| 4019 | 38 | 11 | 10 | The clinician elicits the patient’s preferred level of involvement in decision making.  0 The behaviour is not observed.  **1 A minimal attempt is made to exhibit the behaviour.**  2 The clinician asks the patient about their preferred role in the decision making process.  **3 The behaviour is exhibited to a good standard.**  4 The behaviour is observed and executed to a high standard. | 2  AI = 3  Human = 1 | First of all, I’d like to ask you whether, since our last appointment, you’ve had a chance to think about it, or whether **you have any idea what would work best for you**, or should I go through my possible options with you first? | I’ve already thought about it, but I’m not sure. **I’d like to hear what you suggest** | The physician only **implicitly inquires** about the patient's preferred level of involvement in the decision-making process. The patient replies that he would like to hear the physician's recommendations, thereby providing a **minimal indication** of his preferred level of involvement |
| 4244 | 48 | 10 | 2 | The clinician states that there is more than one way to deal with the identified problem (‘equipoise’).  0 The behaviour is not observed.  1 A minimal attempt is made to exhibit the behaviour.  **2 The clinician conveys the sense that the options are valid and need to be considered in more depth.**  3 The clinician explains ‘equipoise’ in more detail, that options have pros and cons that need to be considered  **4 The behaviour is observed and executed to a high standard.** | 2  AI = 4  Human = 2 | I understand your fear of side effects, and it’s very important to be aware of them, but medication **isn’t the only option.** There are also **various options** in terms of psychotherapy. Have you ever heard of psychoanalytic psychotherapy or cognitive behavioral therapy? | No, I've never heard of that. What is it? | The physician explains that there are **several equally valid approaches**, but does not provide a detailed **overview.** |
